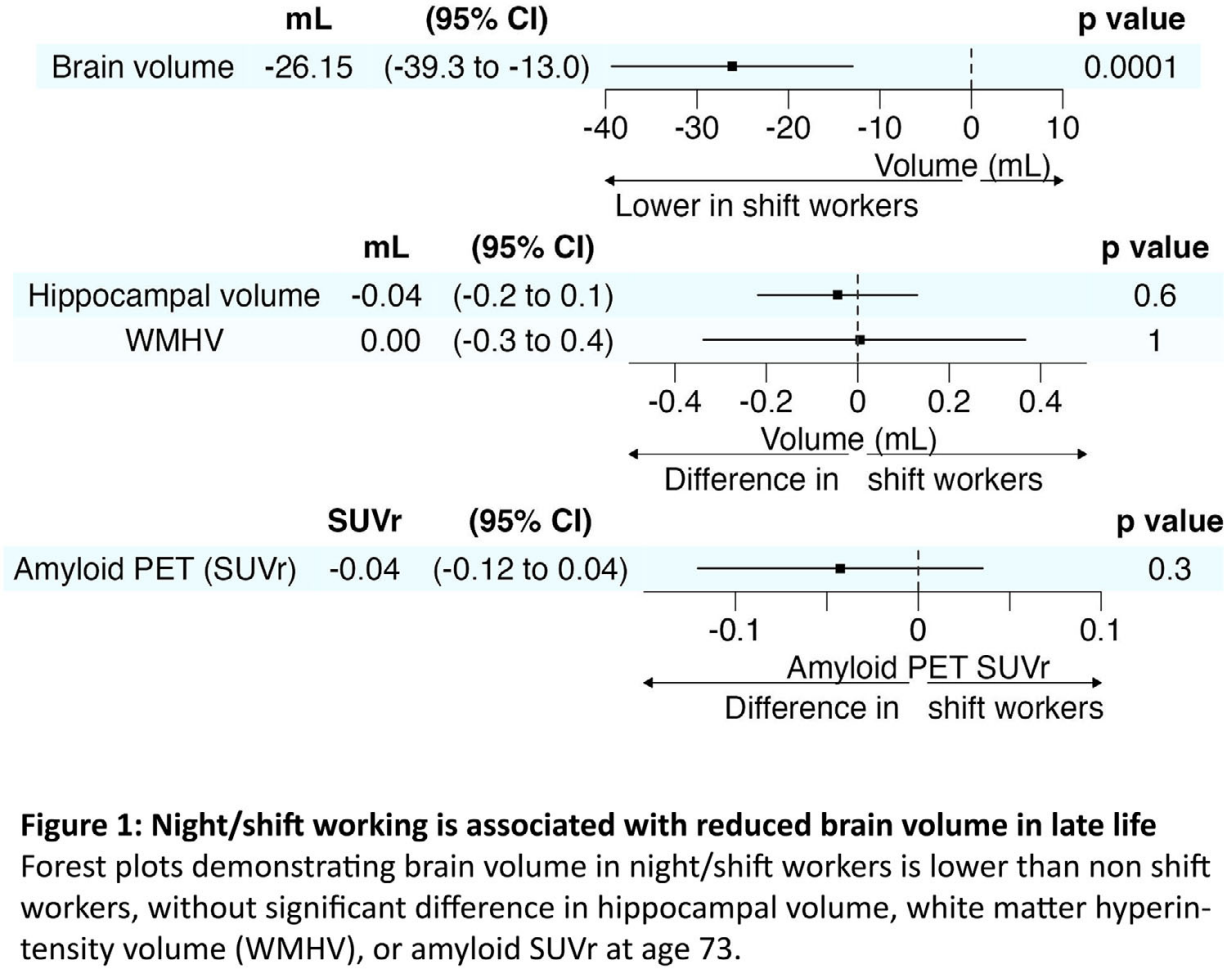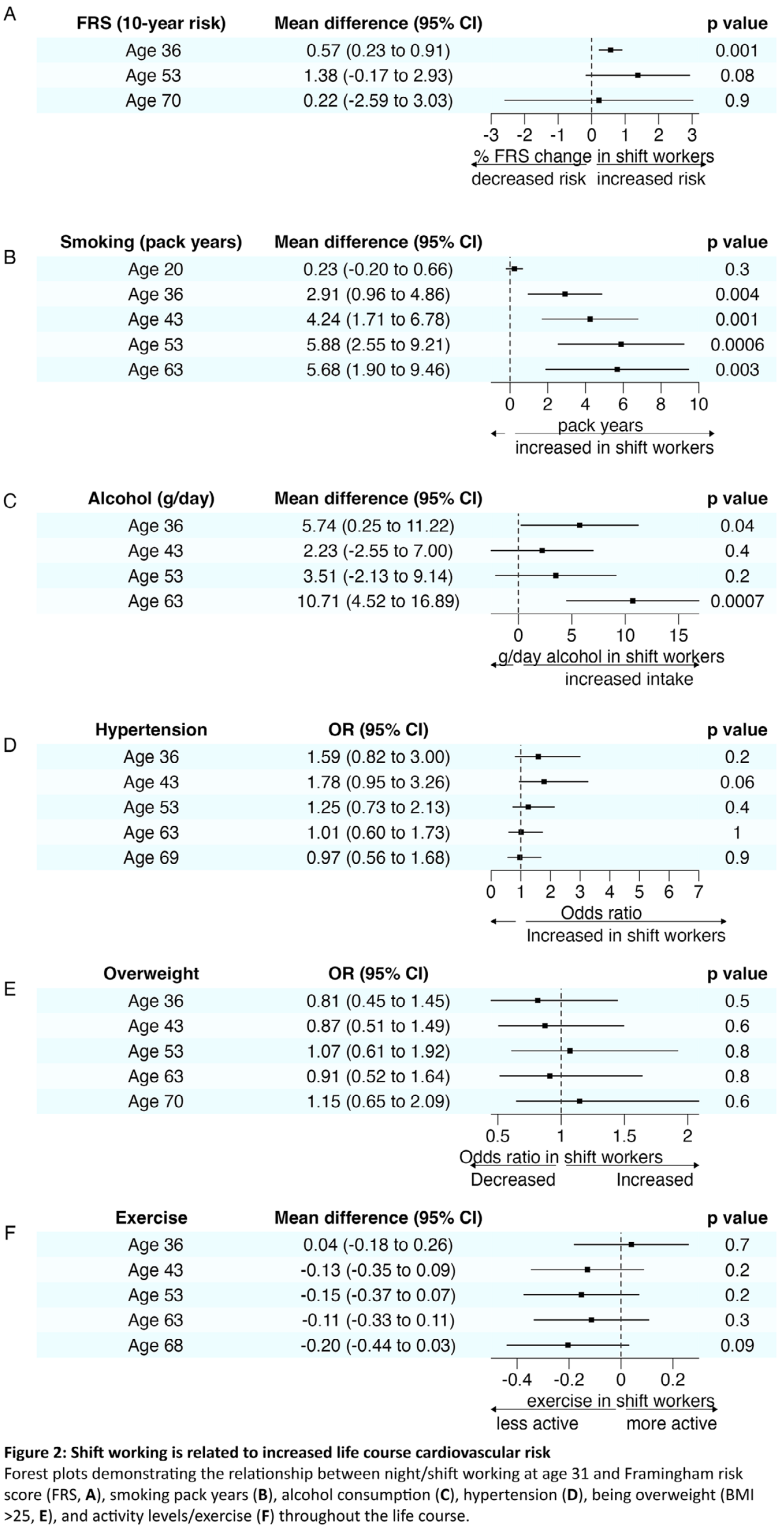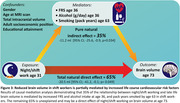# Shift and night work in the fourth decade is associated with reduced brain volume in late life independent of amyloidogenic pathways: an Insight 46 study

**DOI:** 10.1002/alz.092742

**Published:** 2025-01-03

**Authors:** Josh King‐Robson, Jennifer M Nicholas, Sarah‐Naomi James, Carole H Sudre, Kirsty Lu, Jo Barnes, Thomas M. Brown, William Coath, David M Cash, Insight 46 Study Team, Jason D Warren, Marcus Richards, Jonathan M Schott

**Affiliations:** ^1^ Dementia Research Centre, UCL Queen Square Institute of Neurology, University College London, London United Kingdom; ^2^ Department of Medical Statistics, London School of Hygiene and Tropical Medicine, London United Kingdom; ^3^ MRC Unit for Lifelong Health and Ageing at UCL, London United Kingdom

## Abstract

**Background:**

Sleep and circadian disruption are associated with increased dementia risk, yet the mechanism remains poorly understood. We examined the relationship between night/shift working in the fourth decade and late‐life brain health. We explored whether significant relationships were mediated by life course factors including cardiovascular risk.

**Methods:**

Night/shift working (yes/no) was recorded prospectively at age 31. Smoking, alcohol intake, body mass index, exercise, blood pressure, and Framingham risk scores (FRS) were determined at 3‐6 timepoints across the life course (age 20, 36, 43, 53, 60‐64, 68‐70). Whole‐brain and hippocampal volumes, white matter hyperintensity volume (WMHV), and β‐amyloid PET SUVr were derived from T1, fluid‐attenuated inversion recovery, and ^18^F‐Florbetapir PET, respectively, at age ∼73.

Associations between night/shift working, life course cardiovascular risk factors, and imaging metrics were examined with linear regression. Causal mediation analysis (gformula approach in R), examined whether significant relationships between night/shift working and imaging metrics were mediated by life course cardiovascular risk factors. Analyses were adjusted for gender, adult socioeconomic status, educational attainment, age at imaging, and intracranial volume for volumetric measures.

**Results:**

432 participants had available data, of whom 74 (17.1%) were night/shift workers. Night/shift workers had lower whole brain volume (‐26.2 ml, 95% CI ‐39.3, ‐13.0, *P* < 0.001, **Figure 1**), without evidence of a significant difference in hippocampal volume, WMHV, or amyloid‐β SUVr. Night/shift workers had 0.6% higher FRS (95% CI; 0.23%, 0.91%; *P* = 0.001) and higher alcohol consumption age 36 (5.7 g/day, 95% CI, 0.3, 11.2, *P* = 0.04); higher alcohol consumption age 60‐64 (10.7 g/day, 95% CI, 4.5, 16.9; *P* < 0.001); and smoked an additional 5.7 pack‐years by age 60‐64 (95% CI 1.9, 9.4; *P* = 0.003, **Figure 2**). 35% of the brain volume reduction in shift workers was mediated by cardiovascular risk factors (**Figure 3**).

**Conclusion:**

Shift working in the early 30s is associated with lower brain volume in late‐life independent of amyloidogenic pathways. While partially mediated by increased cardiovascular risk factors in night/shift workers, the majority of the effect is unexplained and may be a direct effect of night/shift working on brain health.